# Placing women in Cytogenetics: Lore Zech and the chromosome banding technique

**DOI:** 10.1186/s13039-021-00560-3

**Published:** 2021-08-05

**Authors:** Felicitas Söhner, Nils Hansson

**Affiliations:** Department of the History, Philosophy and Ethics of Medicine, Centre for Health and Society, Faculty of Medicine, University of Düsseldorf, Moorenstr. 5, 40225 Düsseldorf, Germany

**Keywords:** Chromosome painting, Chromosome aberration, Biography, European, Visibility, Recognition, Gender

## Abstract

**Background:**

Scholars agree that Torbjörn Caspersson’s lab at the Institute of Medical Cell Research and Genetics at the Karolinska Institute, Sweden, played a key role in the first description of the so-called Q-banding technique. It laid the foundation for a new era of cytogenetic diagnostics and had a lasting impact in several areas of biology and medicine.

**Methods:**

Based on a mixed-method approach, essential aspects of the history of human cytogenetics are considered via primary and secondary analysis of biographical interviews as well as the qualitative evaluation of bibliometrics. Drawing on interviews with colleagues of lab member Lore Zech (1923–2013) and contemporary publications, this paper illuminates the role of and contribution by Zech: To what extent is the discovery attached to her and what does her legacy look like today?

**Results:**

The analysis of the contemporary witness interviews with colleagues, students and junior researchers shows that Lore Zech was a committed member of Caspersson's research group. In addition, memoirs by contemporary colleagues describe her outstanding skills in microscopy. The different sources paint a multifaceted picture. In addition to the historians' patterns of interpretation, different legacies can also be found within the peer group.

**Conclusions:**

We argue that Zech represent the type of scientist who, although her research was acknowledged with several prizes, so far has not been part of the canon of pioneers of international cytogenetics.

## Introduction

Everyone knows iconographic representations of the chromosome set, but very few in the international research community can trace the origins around the discovery of specific banding patterns on human chromosomes. In the case of the geneticist Lore Zech (1923–2013), the question arises as to whether discrepancies exist in the perception and transmission of scientific quality and professional reputation.^1^ In some contributions on the history of human cytogenetics, the introduction of chromosome banding in humans is attributed to Zech.^2^ In order to illuminate her legacy, it is important to look not only at the written history of the discipline (cultural memory), but also at interviews and memoirs of colleagues and students. This paper takes, for the first time, a closer look at Zech’s reputation in the two countries where she conducted research: Sweden and Germany, and provides a critical re-analysis of her contributions related to the discovery of the so-called Q-banding technique.

Born in 1923 in Gütersloh, Lore (birthname Vogt-Köhne) grew up at the German countryside with her grandparents, after both her parents had passed away due to tuberculosis. Zech first studied medicine in Marburg but changed field and completed a degree biology, chemistry and physics in Bonn. After receiving her doctor diploma at the Max Planck Institute in Tübingen, Zech followed her husband Hendrik Zech to the Karolinska Institute in Solna, Sweden. There, she started her postdoc studies at the Institute of Cell Research and Genetics, headed by geneticist and cytologist Torbjörn Caspersson, in 1953.^3^ At that time, Caspersson had recently published his landmark book *Cell growth and cell function: a cytochemical study* (1950). Zech was engaged in several areas of research in the Caspersson lab in the 1950’s and in the 1960’s,^4^ but the major scientific breakthrough took place in the late 1960’s. Caspersson's and Zech's most cited paper to date has more than 1100 citations (with Caspersson as first author),^5^ which contributed to Zech being among the 1000 most cited contemporary scientists worldwide in the period 1965–1978.^6^

In 1969, Zech developed the first technique for the differential representation of human chromosomes, based on chromosome bands in plant genetics.^7^ With the help of this Q-banding technique, individual chromosomes and their sections, such as chromosomal changes,^8^ could be identified and described on the basis of characteristic band patterns.^9^ This technique received official recognition as early as in 1971 at the 4th International Conference “*Standardization in Human Cytogenetics*” in Paris.^10^ At this conference, the QM fluorescence band pattern (Q-banding) was compared with the Giemsa banding (G-banding) developed in 1971. Even though the so-called G-banding gained the upper hand within less than two years, the priority for human chromosome banding is ascribed to Lore Zech. Since then this technique has been part of the standard repertoire of cytogenetic diagnostics,^11^ and it has been described as a prerequisite for subsequent molecular studies on the human genome and gene-splicing.^12^ Some human geneticists see this discovery as the beginning of a new era in clinical cytogenetics and tumour cytogenetics.^13^

Zech’s breakthrough was recognized with several prizes and honorary memberships, including the German Federal Cross of Merit 1st class. She was the first scientist to receive the Mauro Baschirotto Award by the European Society of Human Genetics; in addition, she was also one of the first scientists to receive the Medal of Honour of the German Society for Human Genetics (GfH). It exists a Lore Zech-Prize, awarded by the German Society for Human Genetics. Still, Zech is not a household name to many researchers in the international scientific community. In addition, she received the honorary doctorate of the Medical Faculty of the Christian-Albrechts-University, Kiel, the Björkén prize of the Medical Faculty of the Uppsala University and the Gunnar Dahlberg-Medal of the Northern Society of Pathology.

During the last decade, historians have dealt with her professional activity and influence. Focusing on research done by women, the Spanish historian María Jesús Santesmases recently stated that the Q-banding technique was established by Lore Zech.^14^ Based on recollections by Lore Zech, Santesmases meant that Caspersson himself had shown little interest in chromosomes and that Zech had largely worked on them by her own.^15^ In the account of the Swedish historian Olof Ljungström, Zech's scientific achievement is rather relativised. He argues that Caspersson worked out the implications of the discovery.^16^

Against this historiographical background, this article brings together several perspectives to further trace the legacy of Lore Zech. How was she portrayed in interviews, in contemporary publications, and what does her status in the scientific community look like today?

## Method

Starting from the previously formulated perspectives of the historiographers and historians of science, this article draws on primary and secondary literature in English, Swedish and German, but also on oral history. The latter method collects biographical accounts and examines them for narrative strands and themes, which are then compared with other source material.^17^ For this paper, a secondary analysis of expert interviews was conducted.^18^ Secondary analysis does not automatically imply that the data used must be interview data from other researchers; rather, data from one's own surveys can also be included in order to address new research questions.^19^

In addition to written memory, the oral history approach offers complementary access to different perspectives. The empirical basis is formed on the one hand by expert interviews conducted by Felicitas Söhner between 2016 and 2018 with human and medical geneticists active between 1970 and 2000.^20^ In the series of 33 recorded interviews, three of the 29 released interviews address developments in cytogenetics and the role of Caspersson's lab (Christa Fonatsch, Jan Murken, Klaus Zang).

Further, recorded expert interviews from an interview series with founders of human and medical genetics conducted by Peter Harper between 2003 and 2014 were included. In Harper's series of 100 recorded interviews with experts, some conversations delve into the beginnings of cytogenetics in the late 1950s and research into its medical genetic application in the 1960s and 1970s (Henry John Evans, Jan Lindsten).^21^ Besides an interview with Lore Zech in 2004,^22^ individual interviews in the series explore the development of the chromosome banding technique and the role of Lore Zech. These provide an exciting foil for comparison with the perspectives of literature and other recorded memories.

These interviews were analysed using qualitative, historical-hermeneutic and social science methods. The approaches of qualitative content analysis according to Mayring^23^ and the so-called Grounded Theory^24^ were applied. Mayring's qualitative content analysis focuses on the ordering, categorisation and structuring of manifest and latent content and the development of systematic and intersubjectively verifiable results.^25^ Grounded theory pursues the goal of developing new theories based on an open question by means of content analysis of interviews, field observations and other empirically collected data.^26^ This mixed-method approach opens new perspectives to the question how Lore Zech was portrayed in Sweden, Germany and in an international perspective.

## Results

The performances of researchers are attributed by the scientific community in various ways. In the academic world, social networks play a significant role; good networking guides^27^ and thus the visibility of academics.^28^ With regard to the visibility of the scientist Lore Zech, the following stood out as particularly prominent in the categories addressed: invitations from or by professional colleagues, attendance at conferences and congresses, involvement in research contexts, classification of scientific achievements.

### Perspectives on Zech by human- and cytogeneticists

Written sources show that Zech was in close contact with German-speaking researchers, including Thomas Cremer, Christa Fonatsch, Simone Heidemann, Anna Jauch, Peter Lichter, Brigitte Schlegelberger and Evelin Schröck.^29^ This is also reflected in the eyewitness interviews of professional colleagues.

The retrospective perception paints a picture of a mentor to numerous young researchers who sought contact through regular letters, invitations and open discussion at conferences. In the recollection of her colleague and mentee Schlegelberger, Zech enjoyed the trust of many and was always surrounded by scholars at conferences. This had contributed to her being valued as an inspiring mentor.^30^ The geneticist Fonatsch also ranked Lore Zech as one of the scientists who had had the greatest influence on her own career: *“and of course the professors (Lore) Zech and Janet Rowley and Margareta Mikkelsen from Denmark… those are the most important ones…”.*^31^

The professional networking went beyond collegial exchange and professional support. It can be seen that Lore Zech's visibility is also evident in the perception of her participation at international conferences and congresses, important arenas for building and participating in networks.

In a biographical review, Schlegelberger pointed out that, in 1971, Zech's achievement was recognised by an invitation to the International Congress of Human Genetics in Paris to present the structure of human chromosomes that she had elucidated with the help of the Q-banding technique. The first version of the international cytogenetic nomenclature ISCN was also established at this conference.^32^

These perspectives are reflected in the oral memories of Lore Zech. The impact of her participation in this congress is also recalled by Zech in the interview with mixed feelings^33^: “*But I had talked about the Y chromosome at the conference in Reykjavik … I had to show Caspersson my manuscript and … he wrote on the big space on the side ‘This is ridiculous. You shouldn’t talk about it. People will laugh at you’. But anyhow, Albert de La Chapelle was there and he became very interested and the next opportunity he came to Stockholm. But when he came to see my chromosomes, Caspersson thought there might be something with them*.”^34^

The memories of witnesses paint a picture of good institutional integration of Lore Zech in Caspersson’s lab and intensive networking with external colleagues and scholars from other disciplines. The biographical documents refer to diverse professional collaborations with Lore Zech, which presumably strengthened the perception of her research.

Schlegelberger underlined the collegial exchange with Janet Rowley, which led to the description of the origin of the so-called “*Philadelphia translocation*”.^35^

The interviewees also mentioned Zech's involvement in the research contexts at the Karolinska Institute. Karolinska geneticist Jan Lindsten, for example, described the joint scientific work and professional interests,^36^ which were quite different from Caspersson's: “*Now even that became a problem after some time, because Caspersson was so fascinated by machines and measurements and he made very good machines. He was very good at that. … He was interested in measuring in itself, which is legitimate I must say, but that was not my interest. So Lore and I had so many problems we wanted to do, but Caspersson more or less prevented us from pursuing all these ideas we had.*”^37^ Lindsten also addressed how Caspersson viewed their collaboration: “*… but as Lore and I wanted to continue we had so many ideas of what to do, but he stopped it more or less. Of course I could continue with the banding techniques myself, but he stopped the collaboration between me and Lore because it became tangled. It grew too fast and too much and he hadn’t any control over it.*”^38^

In addition to good interdisciplinary networking, international, intradisciplinary networking was also essential for the visibility of the research results. Thus, in Zech's recollection, in addition to the presentation of current results at conferences, the personal report of directly involved people to colleagues in other institutes also contributed to the popularity of their results: “*We saw the Y chromosome and then we had real luck, because one of our students at the Institute, who is now a professor in Lund [Sweden], he travelled to Germany and told everywhere, in our Institute they have very interesting methods to get bands on chromosomes and they can see the human Y chromosome, and so on and so on. But nobody used this.*”^39^

Caspersson and Zech have repeatedly been described as key figures in the discovery of banding in human chromosomes, a view shared by Caspersson himself and Zech's long-time Karolinska Institute colleague Gösta Gahrton. But how is Zech's scientific achievement classified in the respective historical retrospectives?

The director of the institute Caspersson himself introduced her in 1989 “*my long-time collaboratress Dr. Lore Zech*.”^40^ Talking about Zech’s role, her lab colleague and later chairman of the Nobel Committee of physiology or medicine Gösta Gahrton phrased it as follows (translated from Swedish): “*Who first discovered the bands is unclear, maybe Evy Simonsson, maybe Lore Zech, but probably it was Lore who saw that two plant chromosomes had band patterns that seemed identical while other chromosomes had other band patterns.*”^41^ Harper, who has conducted numerous biographical interviews, assigns Zech the following role: “*Zech's pioneering discovery as employee of Caspersson's laboratory*”.^42^

Furthermore, in the interview her colleague Henry John Evans (Edinburgh) described an unequal distribution of work that he perceived. In his memory, Zech did not work under him, but together with him – and she had the main workload: “*At that time Torbjorn Caspersson had just begun to start getting somewhere with fluorescence microscopy and using quinacrine, and Lore Zech worked with him. Lore, she did all the work actually, but for Tjorbjörn [Caspersson] everything had to be done with a spectrometer*.”^43^ In the biographical interview, Zech herself rates her discovery as “*very outstanding*”.^44^

In the interview, Jan Murken assessed the discovery of “*Zech and Caspersson*”^45^ as a groundbreaking methodological paradigm shift in human genetics. In his review of the history of the discipline, Murken refers to the “*banding technique of Lore Zech*”.^46^

Looking back on Caspersson's role, she referred several times to the fact that Caspersson did not believe in the existence of the banding at first, but understood the weight of the discovery.^47^ Elsewhere in the interview, Zech again stated that he had little confidence in the banding technique.^48^

In the obituary by Schlegelberger it is described that Zech was a collaborator “*under*” Caspersson, who nevertheless worked “*independently*” on this development and thus achieved a scientific breakthrough.^49^ Schlegelberger underlined Lore Zech's essential role and emphasised her pioneering role as the „*mother of modern cytogenetics* “.^50^

In conclusion, the perceptions and interpretations of geneticists appear heterogeneous. Here we find both statements that downplay Zech's achievements and passages that praise her.

## Zech’s scientific publications

According to Siebert, researchers who are rarely cited have only a very small chance of coming to the attention of other scientists.^51^ So what about Lore Zech's reputation? Did she receive the same recognition as her male colleagues? We systematically reconstructed Zech’s publications in the journals in which she has published most of her articles, “Experimental Cell Research” and “Hereditas”. The editors of this journals were scientists from Scandinavia.

**Fig. 1:**
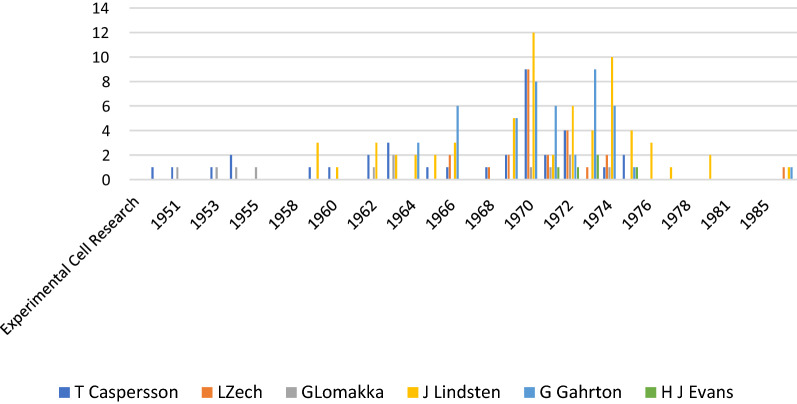
Articles in Exp Cell Research 1950–1988. The number of articles per year by Zech and the co-authors involved in the period 1950 (first appearance of the journal) to 1988 (last publication by Zech in the journal) in the journal Experimental Cell Research

The quantitative comparison in “Experimental Cell Research” that has published articles from the field of cell biology since 1950 shows that she published in that forum on a regular basis (Fig. 1). It is shown that the number of Zech's publications shows a peak in the seventies. In this journal alone there are 13 contributions by Caspersson and Zech in the years around 1970 and 1972. Besides Caspersson, Lindsten, Evans and Gahrton, these include above all Gösta Lomakka, Albert Levan, Maj Hultén, Edward J Modest, Richard Buckland, Evy Simonsson, Adrian T Sumner, C Johansson and Joe Hin Tijo.

**Fig. 2:**
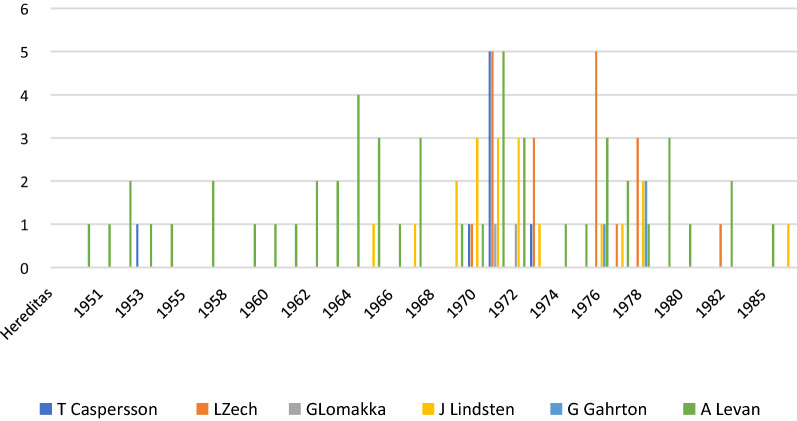
Articles in Hereditas 1950–1988. The number of contributions per year by Zech and the co-authors involved during the same period in the journal Hereditas

The quantitative view of the contributions in the journal Hereditas reveals another phase of clustered publications by Lore Zech in the late 1970s and early 1980s (Fig. 2). The fact that Lore Zech rarely appeared as a first author in her young career indicate her reputational differences over time.

A look at Hereditas shows that works with Caspersson as first or co-author appear much less there. Works by Zech appeared there not only in the phase shortly after the discovery of the banding technique (1970–1973), but also several years later.

A look at Zech's bibliography shows that she only appeared as first author every sixth time and more frequently as co-author. At the same time, she published repeatedly in journals of higher impact with different subject foci and together with renowned co-authors. It is noticeable that all of Lore Zech's papers from Casperson's laboratory were written by Caspersson as first author.

It also gives the impression that the question of arrangement was consistently justified by alphabetical order.

Several sources and reports of contemporary witnesses substantiate the conclusion that that all papers from Caspersons labs are first authored by him because he published usually with alphabetical list of all authors and never had an employee starting with last name A, B or C. The question of the effect of alphabetical order on the careers of scholars was discussed in the late 1970s.^52^

## Conclusion: Zech’s legacy today

It is important to look into how excellence has been attributed to individual scientists to deepen our understanding of reward mechanisms in medicine. By reconstructing views on Zech’s scientific achievements and reputation, this article aims at stimulating a discussion about the recognition of female scholars in genetics and beyond.

Scholars agree that the team of Caspersson in general and Zech in particular played key roles in the discovery of the Q-banding technique, which became an important diagnostic tool and also provided a means to unravel pathogenetic mechanisms by pinpointing the location of cancer-initiating genes. It laid the foundation for a new era of cytogenetic diagnostics and had a lasting impact in several areas of biology and medicine. Even though Q-banding was superseded within a few years by G-banding, which was developed in 1971, the priority for human chromosome banding lies with Lore Zech.

It should be noted that Zech developed the Q-banding method as an employee of Caspersson's laboratory. At that time, it was common for the head of an institute to claim the research results for himself; even today, in some disciplines, group or institute leaders are listed as first or last authors by default. These observations are also set against the backdrop of a time when it was considered common that researchers in the second row of a research team did not get the attention they deserved. Lore Zech was one of them.

Against this background, we considered the question of how to assess Zech's visibility in the national and international research community and how to evaluate the memory of her role in the biographical documents and scientific publications. The analysis of the contemporary witness interviews with colleagues, students and junior researchers shows that Lore Zech was a committed member of Caspersson's research group. In addition, memoirs by contemporary colleagues describe her outstanding skills in microscopy.

That said, in these accounts there exist differences in the culture of remembrance.

The different sources paint a multifaceted picture. In addition to the historians' patterns of interpretation, different legacies can also be found within the peer group. They range from relativising her findings to describing her as „*mother of modern cytogenetics* “.^53^ The biographical reports show that Lore Zech was not only perceived as a mentee. Rather, the interviewees recall her commitment on a broader level as a networker, promoter and mentor.

On a more general level, we argue that such a mixed-method presented here via primary and secondary analysis of biographical interviews such as the qualitative evaluation of bibliometrics offers a rewarding concept for historical analysis not only of the history of human cytogenetics, but also for other areas in the history of medicine and science.

## Data Availability

The datasets used and/or analysed during the current study are available from the corresponding author on reasonable request.

## Footnotes

1 Santesmases [1], 395–430. Cf. 414, 428; [2], Cf. 103f).

2 Hsu [3]: 100 f.

3 Hansson [4].

4 Zech [5–7].

5 Caspersson et al. [8].

6 Garfield [9].

7 Caspersson [10, 8].

8 e.g. the Burkitt translocation t(8;14).

9 Borkhardt [11].

10 Hamerton et al. [12] [Chromosome: Banding techniques: 320–332].

11 Murken et al. [13].

12 Ljungström [14].

13 Ruckpaul et al. [15].

14 Santesmases [16].

15 Santesmases [16]: 189.

16 [Original Swedish] “Det kom att bli i första hand Casperssons docent, Lore Zech senare professor, som utarbetade implikationerna av upptäckten. Värdet av den är att den för första gången gjorde varje enskild kromosom i det mänskliga genomet tydlig och konsekvent urskiljbar.” Upptäckten brukar beskrivas som ett av de första stegen på vägen till utvecklingen för”gene-splicing” Ljungström [14, pp. 245–246].

17 Plato [17].

18 Söhner [18, 19].

19 Heaton [20].

20 Söhner [21].

21 Harper [22].

22 Zech [23].

23 Mayring [24].

24 Strauss and Corbin [25].

25 Mayring [24].

26 Strauss and Corbin [25].

27 Brothun [26].

28 Bucchi and Trench [27].

29 Schlegelberger [28].

30 Schlegelberger B: In memoriam: Prof. Dr. rer. nat. Dr. med. h.c. Lore Zech; 24.9.1923–13.3.2013: Honorary member of the European Society of Human Genetics, Honorary member of the German Society of Human Genetics, Doctor laureate, the University of Kiel, Germany. Mol Cytogenet 2013, 6:20.

31 Fonatsch and Söhner [29].

32 Schlegelberger [28].

33 Zech and Harper [23].

34 Zech and Harper [23].

35 Schlegelberger and Söhner [30].

36 Lindsten [31].

37 Lindsten and Harper 32.

38 Lindsten and Harper [32].

39 Zech and Harper [23].

40 Caspersson [33].

41 [Original Swedish] “Vem som först upptäckte banden är oklart, kanske Evy Simonsson, kanske Lore Zech, men troligtvis var det Lore som såg att två växtkromosomer hade bandmönster som verkade identiska medan övriga kromosompar hade andra bandmönster.” Gahrton G. I Nobelprisets skugga. Stockholm 2019.

42 Harper [34].

43 Evans and Harper [35].

44 Zech and Harper [23].

45 Murken and Söhner [36].

46 Murken [37].

47 Zech and Harper [23].

48 Zech and Harper [23].

49 Schlegelberger [38].

50 Schlegelberger [38].

51 Siebert [39].

52 Rudd [40].

53 Schlegelberger (2013).
